# Functional predictors of treatment induced diabetic neuropathy (TIND): a prospective pilot study using clinical and neurophysiological functional tests

**DOI:** 10.1186/s13098-022-00805-0

**Published:** 2022-03-03

**Authors:** Yvonne Hoffmann, Klaus V. Toyka, Matthias Blüher, Joseph Classen, Petra Baum

**Affiliations:** 1 Department of Neurology, University of Leipzig, Liebigstraße 20, 04103 Leipzig, Germany; 2 Department of Neurology, University of Würzburg, Josef-Schneider-Str. 11, 97080 Würzburg, Germany; 3 Department of Medicine, University of Leipzig, Liebigstraße 21, 04103 Leipzig, Germany

**Keywords:** Treatment-induced neuropathy of diabetes (TIND), Diabetes mellitus, Heart rate variability, 30:15 ratio, Neuropathic pain, Autonomic neuropathies, HbA1c

## Abstract

**Background:**

A treatment-induced drop in HbA1c has been suggested to be a risk factor for TIND.

**Methods:**

From 60 included patients with severe diabetes mellitus (HbA1c over 8.5) only 21 patients adhered to the study protocol over 1 year with a battery of autonomic nervous system tests scheduled before and after starting antidiabetic treatment.

**Results:**

In patients with a drop of HbA1c greater than 2 per cent points only some neurophysiologic tests and lab values tended to deteriorate with a trend to improve at later time points along the study. None of these changes were statistically significant, most likely because the study failed to reach the planned number of patients.

**Conclusion:**

Poor adherence to diabetes treatment and to following the study protocol were the assumed obstacles in our patient cohort selected for very high HbA1c levels. In future studies a multi-center trial and case numbers of up to 500 patients may be needed to account for drop outs in the range observed here. Moreover, the number of tests in each patient at each visit may have to be reduced and special educational group sessions are warranted to cope with the limited adherence.

*Trial registration* Ethic Committee University of Leipzig 439/15-ek. Registered 22 April 2016

## Background

Treatment-induced neuropathy of diabetes (TIND) is a subacute type of diabetic neuropathy affecting small peripheral nerve fibers [1, 2]. TIND is characterized by acute neuropathic pain and autonomic dysfunction starting within 8 weeks of therapy initiation [3, 4]. Concomitant rapid decrease in HbA1c of more than 2 percent points over 3 months is usually seen [2, 5–7]. In a retrospective study with a 5-year observation period, Gibbons and Freeman [5] found that 11% of patients with diabetes developed TIND. The pathogenic relevance of a fast decline in HbA1c for the manifestation of TIND has been corroborated in a rodent diabetes model [8].

Originally, TIND was treated by reducing insulin doses tolerating a permissive hyperglycemic metabolic state [5, 9]. Reports of the efficacy of this treatment regimen were rarely documented [5]. Yet, maintaining chronic hyperglycemia may in itself augment the well-known long-term complications [10]. The present treatment recommendations including insulin and other anti-diabetic compounds aim at a slower and gradual decline of HbA1c levels [11].

Under appropriate treatment TIND is a self-limiting disorder lasting over weeks or several months [3, 9, 11].

Symptomatic treatment of severe neuropathic pain includes antiepileptic drugs like pregabalin, tricyclic antidepressants, and in cardiac autonomic neuropathy angio-converting enzyme inhibitors or antiarrhythmica [5, 7].

One of the potentially life-threatening complications of TIND-associated autonomic neuropathy is arrythmia and cardiac failure that may lead to increased mortality [12].

Prospective studies are needed to investigate potential predictive factors for developing TIND including associated autonomic neuropathy. We therefore, initiated a single center, prospective pilot study in patients with diabetes and baseline HbA1c levels above 8.5%. At baseline and after receiving adequate treatments over a period of 1 year, we aimed at detecting abnormalities in various tests of autonomic dysfunction that might predict the risk for TIND by utilizing non-invasive neurophysiological functional test procedures.

## Patients and methods

Sixty patients (23 women, 37 men) were screened when diagnosed with diabetes mellitus type 1 or 2 and HbA1c values greater than 8.5%. Out of these, 21 patients (5 women, 16 men) agreed to be repeatedly examined over a period of one year. Clinical and neurophysiological examinations were planned for all patients at baseline (T0) and after 3 (T1), 6 (T2), and 12 months (T3). In addition to standard blood analyses including HbA1c we performed measurements of C-reactive protein (CRP) to detect inflammatory pathology. A battery of non-invasive neurophysiological function tests was conducted including the following tests procedures: cardiovascular autonomic reflex tests (30:15-ratio; Valsalva-ratio, E/I-ratio); examination of heart rate variability during standing or deep breathing [2, 13]; sympathetic skin responses (SSR) inducing the sympathetic skin reflex by an electrical stimulus) [13]; pupillography upon presenting a light stimulus; measuring pupil diameter in darkness (PDD) as an early indicator of autonomic neuropathy [14]; thermography defining a cold/warm perception threshold (CPT/WPT) as a robust indicator of small (C-fibers) and of larger, myelinated A delta fiber pathology [15]; quantitative sudorimotor axon reflex tests (QSART) measuring sweat production after stimulation with acetylcholine [13]; Collectively, the group of cardiovascular autonomic reflex and the QSART examine parasymphathetic function while PDD and the other autonomic tests examine sympathetic function [2, 14]. Abnormalities in any of these procedures indicate small fiber neuropathy. The study protocol was approved by the Ethics Committee at our institution (No. 241-2009-0911209). All participants gave written informed consent. The differences of the HbA1c values and the differences of the neurophysiological tests between T0 and T1 were calculated and used for Pearson’s correlation analyses. Patients were grouped according to the treatment-related reduction of HbA1c. Group A consisted of patients whose HbA1c dropped by 2 percentage points or more and group B dropped by less than 2 percentage points. Comparisons between the groups and between points in time were analyzed using unpaired (groups) and paired (time) t-tests and Wilcoxon–Mann–Whitney tests [16]. SPSS version 16 was used for the statistical analyses. We expected deterioration in neurophysiological test results at T1 as compared to T0, with further deterioration during the subsequent course. All participants gave written informed consent.

## Results

Of the screened 60 Patients, only twenty-one (35%) agreed to participate in the planned further test procedures. Of the 21 only 13 achieved an HbA1c value reduction of more than 2 percent points in the first 3 months. Overall adherence was 22%.

The 21 patients participating over 1 year received a full battery of tests while receiving effective anti-diabetic medication and dietary recommendations. In the 13 patients of group A, mean reduction in HbA1c from T0 at T1 was 4.8 percent points (p = 0.001). Of these, only one patient suffered from neuropathic pain at T1 which later regressed. In the 8 patients of group B mean reduction of HbA1c was only 0.13 percent points on average (p = 0.53) suggesting an insufficient choice of anti-diabetic treatment or, more likely, poor treatment adherence (Table 1).

**Table 1:** Baseline characteristics

	All patients (n = 21)	Group A (n = 13)	Group B (n = 8)	p value
Male sex	16 (73%)	10 (77%)	6 (67%)	0.6*
Age (years)	49.9 ± 3.7	50.5 ± 3.9	49.1 ± 7.2	1*
HbA1c (%) at T0	10.5 ± 1.7	11.2 ± 1.6	9.4 ± 1	0.03*
HbA1c (%) at T1	7.4 ± 0.4	6.4 ± 0.3	9.2 ± 1.1	0.01*
Difference T0–T1	3 ± 2.9	4.8 ± 3	0.1 ± 1.1	< 0.001*
Duration of diabetes	8.5 years	0.23 years Minimum 0 Maximum 2	20 years Minimum 8 Maximum 57	< 0.001*

*Comparisons between group A and B. p value calculated according to Mann–Withney-U-Test; siginificant values are marked bold

**Fig. 1:**
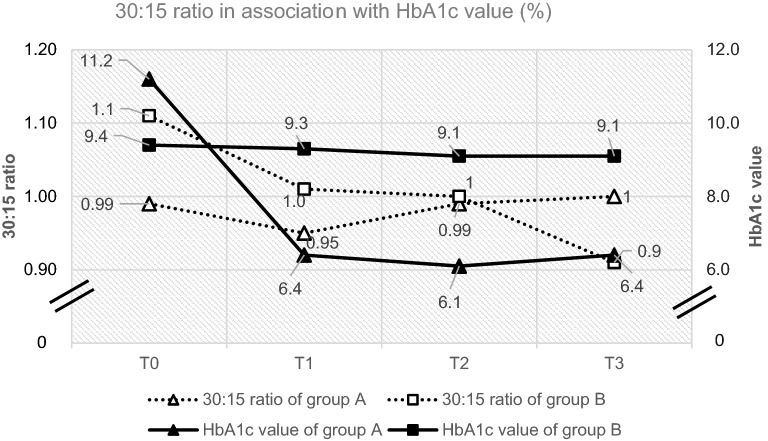
30:15 ratio of group A and B over 1 year. Group A: HbA1c values improved by 4.8 percentage points at T1 and leveled off at around 6.3% during the course. 30:15 ratios tended to deteriorate after 3 months (T1) and subsequently improved over the following 9 months. Group B: HbA1c values exceeded those of group A at all time points after treatment initiation. 30:15 ratio deteriorated continuously over a year.

While values of functional tests were quite similar at baseline (T0) in both groups, patients of group A tended to later display abnormal test results in the 30:15 ratio, E/I-ratio, and CPT at T1. Subsequently, these abnormalities regressed. In contrast, in group B the test results tended to gradually deteriorate over 1 year. As an example, the course of the 30:15 ratio data is shown over 1 year (Fig. 1).

Furthermore, thirteen participants already showed clinical signs of diabetic sensory neuropathy at T0 (e.g., reduced vibration threshold), which was finally interpreted as a consequence of a hyperglycemic metabolic state. We followed these patients with overt neuropathy and compared the functional data over the individual study period (see Table 2, especially NES, CPT).

**Table 2:** Electrophysical parameters of group A and B and correlation analysis of all participants

	T0	T1	p value	T3	p value
30:15 ratio	1 ± 0.12 (1.1 ± 0.3)	0.95 ± 0.1 (1 ± 0.2)	0.12 (0.8)	1 ± 0.13 (0.9 ± 0.2)	0.62 (0.3)
E/I ratio	2.91 ± 2.7 (2.6 ± 1)	2 ± 0.9 (2.6 ± 0.7)	0.18*** (0.8)	2.4 ± 0.9 (2.7 ± 1)	0.65 (0.3)
PDD (mm)	5.6 ± 0.5 (5.4 ± 1.7)	5 ± 1.3 (5.3 ± 1.5)	**0.02** (0.6)	5.4 ± 1 (5.4 ± 1.5)	0.3 (0.6)
CPT (°C)	27.3 ± 3 (22 ± 9)	25.1 ± 5.9 (24 ± 3.2)	0.1*** (0.4***)	26.2 ± 4.5 (20 ± 10)	0.4*** (0.6***)
NES	2.8 ± 2.3 (4.1 ± 4)	1.2 ± 1.3 (2.4 ± 2.3)	0.04 (0.2)	1.8 ± 1.9 (3.1 ± 2.9)	0.2 (0.7)
NSS	4 ± 5 (4.5 ± 5)	3.2 ± 5 (10.9 ± 11)	0.3 (0.08)	4.8 ± 8 (11.2 ± 15)	0.5 (0.2)

Correlation analysis					
Difference between T0 and T1		Pearson correlation coefficient		p value	
30:15 ratio		0.121		0.6	
Valsalva ratio		0.02		0.94	
E/I ratio		0.4		0.08	
PDD		0.2		0.45	
CPT		0.21		0.37	
WPT		0.06		0.81	
Latency of SSR right hand		− 0.16		0.5	
Sweat rate		− 0.034		0.9	

**Comparisons between T0 and T3

p-value according to Wilcoxon-test, ***t-test

When looking at the duration of diabetes in the two groups, it became obvious that group A consisted of newly diagnosed diabetics (0.23 years; one long-standing outlier not included). In contrast, in group B the average duration was 20 years (maximum 57 years). Two patients from group A had been diagnosed because of ketoacidotic coma. Due to the shorter duration of diabetes a lesser degree of chronic nerve damage was expected in group A. In group B with more expected chronic nerve damage we looked at the progression of neuropathy over the study period (Table 2).

## Discussion

Our study aimed at testing the hypothesis that a rapid, treatment induced reduction of HbA1c may be associated with a more common and pronounced induction of TIND in patients with severe diabetes of type 1 or 2 (HbA1c > 8.5%). Deterioration of any one test parameter between T0 and T1 in patients with a rapidly lowered HbA1c was considered a candidate becoming a predictor of TIND. Against our expectation we found at most trends but no significant differences in any of the performed neurophysiological tests over time when comparing group A with group B. Within group A that was presumed to develop TIND, we could not reveal any major distinctive patterns of abnormality despite patients’ HbA1c rapidly declining upon treatment initiation. However, the low adherence to the planned study procedures prevented us from obtaining a sufficient number of observations to formally confirm or refute our hypothesis.

We are aware that the major difference, in the duration of diabetes between groups A, i.e. a few months vs. several years in group B may be an important factor influencing the observations reported here. This relates both to the underlying chronic neuropathy state and to the poor treatment efficacy over long periods of time in group B. Patients in this group may more commonly show poor adherence to adequate treatment and dietary behavior and may consequently also be candidates for poor study compliance as noted here.

Our screened 60 patients were all informed and trained how to comply with the treatments and tests. Before each visit, there was a telephone reminder (as effective means of increasing adherence) [17] of the examination date 1 week in advance. When asked for their reasons for discontinuing study participation, patients indicated that they rather wanted to focus on treating their diabetes than participating in study tests. Other reasons were concomitant illness, distance to the study site and that the many tests were too time consuming and even annoying.

One additional reason for non-adherence may be the lack of having structured educational group sessions to support adherence. Still, it has not formally been shown in our German patient population that measures like group sessions would dependably improve adherence [18]. It remains to be tested if adherence would have been substantially better even with regular structured sessions.

The observations in Group A patients are consistent with but no proof of the involvement of parasympathetic and sympathetic C- and A-delta fibers as a potential indicator of treatment-related small fiber neuropathy [1]. Only one patient of group A developed a painful clinical episode over the first 3 months which may be taken as an indicator of mild TIND.

This futile pilot trial underscores the problem of poor adherence and treatment compliance in patients with very high HbA1c values [19]. Moreover, reduced adherence to prescribed medication may also be associated with poor motivation to follow the test protocol which involved a number of visits. Given the observed standard deviation of 0.16 [20] at T1 in our present pooled data, a future TIND trial would need to enroll up to 500 patients to detect a minimal clinically important difference (MCID) at a power of 80%. Any future trial with this number of patients would warrant a multi-center design and should include educational training sessions and more incentives to increase adherence.

As a result of the reduced compliance demonstrated in this pilot study, especially in patients with long duration of diabetes, a follow-up study should include newly diagnosed diabetics who are also likely to have no or less pre-existing neuropathy.

## Conclusions

In conclusion, the observations in Group A patients are consistent with involvement of parasympathetic and sympathetic C- and A-delta fibres as a potential indicator of treatment-related small fiber neuropathy. Because of the inability to recruit and motivate participants autonomic nervous system predictors for TIND can ultimately not be defined. Therefore, a multi-center design effort is encouraged for achieving a high number of patients.

## Data Availability

The sets of data generated during the current study are not publicly available due to privacy regulations but are available from the corresponding author on reasonable request.
